# Improvement of common variable immunodeficiency using embryonic stem cell therapy in a patient with lyme disease: a clinical case report

**DOI:** 10.1002/ccr3.1556

**Published:** 2018-05-02

**Authors:** Richard Horowitz, Phyllis R. Freeman

**Affiliations:** ^1^ HHS Tickborne Disease Working Group Washington D.C. USA; ^2^ Hudson Valley Healing Arts Center 4232 Albany Post Road Hyde Park New York 12538

**Keywords:** *Borrelia burgdorferi*, common variable immunodeficiency, human embryonic stem cell therapy, Lyme disease, post‐treatment Lyme disease syndrome

## Abstract

Bone marrow transplantation and stem cell therapies have been used for the treatment of common variable immunodeficiency (CVID) and other life‐threatening medical disorders. This is the first known case report in the medical literature describing improvement of both Lyme disease and CVID with human embryonic stem cell therapy.

## Introduction

Lyme disease is the most common vector‐borne illness in the United States and Europe, as migratory birds, among other factors, are spreading infections, increasing the burden of illness 1, 2. In 2015, CDC researchers reported an estimated 329,000 new cases of Lyme disease in the United States 3, with a 320% increase in the number of counties affected 4. Multi‐systemic symptoms include fevers, fatigue, musculoskeletal, and nerve pain which may be migratory in nature 5, cardiovascular and neuropsychiatric symptoms with cognitive difficulties, and insomnia 6. The severity of clinical manifestations is often explained by HLA status and autoimmunity 7, differences in Borrelia genotypes 8, associated co‐infections 9, underlying medical problems increasing inflammation 10, 11 and immune responsiveness 12. Common variable immune deficiency (CVID) is the most common immunodeficiency syndrome 13, which is often associated with autoimmune phenomenon 14 and when it is present in patients with Lyme disease and associated bacterial infections, might increase the severity of their illness.

Immunological and autoimmune manifestations are often associated with *Borrelia* infection. These include autoantibody production with production of antinuclear antibodies and rheumatoid factors 15, 16, anti‐myelin 17 and antiganglioside antibodies 18 associated molecular mimicry against the myelin sheath surrounding nerves leading to demyelination 19, and production of inflammatory mediators including chemokines (CXCL9, CXCL10, CCL 13, CCL19 20, and cytokines (TNF alpha, IL‐1, IL‐6, IL‐8, IL‐10, IL‐17, interferon gamma) 21, 22. These may have associated immunomodulatory and immunosuppressive effects. In particular, increased interferon gamma production 23 has been shown to result from Th1 polarization post‐infection with *Borrelia burgdorferi* contributing to increased pathogenesis 24 and autoimmune reactions 25. Autoimmune phenomena have also recently been linked to environmental toxin exposure, including heavy metals, BPA, asbestos 26, and small particle pollution 27, while mold toxin exposure has been reported to be associated with chronic fatigue syndrome and immunosuppression 28. Simultaneous overlapping infections 29, 30 toxins 31 and genetic factors 7 may, therefore, account for resistant chronic illness in patients who manifest with chronic Lyme disease/PTLDS 32. Addressing all the above factors may be necessary to achieve maximum clinical improvement.

Borrelia's effect on the adaptive and innate immune system is in part a result its ability to evade the immune system through hiding in protected niches 33 such as biofilms 34 and the intracellular compartment 35. Cystic forms 36 and intracellular forms with bleb formation 37, 38 may also increase the inflammatory response, while borrelia may avoid immune recognition through frequent recombination of variable surface protein E (VlsE) 39 and inhibition of complement‐mediated lysis 40, 41.

Another factor which plays a role in Borrelia's pathogenesis is the bacteria's ability to suppress long‐lived humoral immunity. Deficiencies of cellular and humoral immunity during Borrelia infection had been previously noted in the scientific literature 42 as antibodies often waned after infection with a prolonged IgM response 43 Baumgarth and colleagues recently reported that *Borrelia burgdorferi* rapidly targets lymph nodes and adversely affects germinal centers, which are required for the development of long‐lived plasma cells and continuous antibody secretion 44. Low IgG levels and subclasses may subsequently result, impairing the ability to effectively treat Lyme and associated bacterial infections.

## Case History

An 18‐year‐old white male with a past medical history significant for multiple infections including Lyme disease, Mycoplasma, Klebsiella, recurrent Staphylococcus infections, EBV, and HHV‐6 presented to our medical office with the chief complaint**s** of moderate fatigue, sore throats that would come and go, frequent sinusitis, diarrhea once a month, back stiffness and neck pain, mild tremors of the hands, insomnia**,** and moderate cognitive difficulties. The patient had developmental delays at age one after being sick with a rotavirus infection, which subsequently led to a malabsorption syndrome with rickets and vitamin D deficiency. Celiac disease was subsequently diagnosed at age 4 along with CVID. He was found to be HLA DQ8 positive with IgA deficiency (frequently associated with celiac disease), but the diagnosis of CVID was made only after the patient had frequent childhood illnesses. These included whooping cough, coxsackie infections, and frequent episodes of sinusitis secondary to Staphylococcus, which spread one time throughout his body, causing a disseminated staph folliculitis. He was started on IVIG at age 4 with Gammunex 80 g IV one time per month along with Isoprinosine, an immunomodulator used for viral infections. The Isoprinosine was stopped after 2 years secondary to neutropenia. At age 5, his illness progressed as he was diagnosed with inflammatory bowel disease and placed on sulfasalazine without clinical help. His malabsorption continued with ongoing diarrhea, although a gluten‐free diet and vitamins eventually helped stabilize GI symptoms. He continued to be severely weak until age 6 requiring physical therapy and occupational therapy, and his health mildly improved by age 7 along with IVIG, vitamins, and diet. Hyperbaric oxygen therapy with GcMAF was also tried during that time without significant benefit.

At age 8, he was diagnosed with PANDAS after a sore throat. He had severe neuropsychiatric reactions with aggressiveness and OCD tendencies, and his pediatrician increased his dose of IVIG along with steroids and placed him on daily Zithromax for 3 months. This improved his clinical symptomatology, but his OCD would flare up after an acute infection and he also suffered from associated symptoms of ADHD, requiring use of Adderall. This led to a workup to determine whether there were any other overlapping factors accounting for his neuropsychiatric symptoms. He was found to have elevated levels of heavy metals including lead, mercury, and aluminum, as well as black mold, resulting from toxic mold exposure at his middle school. He took phosphatidylcholine, glutathione, and WelChol (colesevelam) for the mold along with a BEG spray (Bacitracin, EDTA, gentamycin), as well as IV EDTA on and off for 10 years for his heavy metals. His metals and mold levels remained elevated however on subsequent testing. The most recent provoked urine heavy metal test using DMSA showed an elevated level of lead at 7.6 (normal is less than 2), a borderline elevated mercury level at 2.9 (normal is less than 3), and urine mold testing showed elevated levels of Ochratoxin A at 6.58500 ppb (normal is less than 1.8 ppb), Trichothecenes at 0.53300 (normal is less than 0.18 ppb), and gliotoxins at 2.60500 ppb (normal is less than 0.5 ppb).

At age 9, he had a tick bite with negative Lyme testing, but his Lyme test turned positive one year later in 2010, with a CDC positive IgM Western blot. The diagnosis was questioned due to the possibility that Lyme antibodies were present in his IVIG, although clinically the patient felt better on cephalosporins. His pediatrician placed him on IV Rocephin for 4 months at 1 g twice a day, 5 days per week. He developed back pain during the treatment which then resolved, and his memory and concentration significantly improved on antibiotics, helping to confirm the diagnosis. Despite being HLA DR 4 positive (associated with increased autoimmune reactions with Lyme disease), he tolerated the protocol well.

Associated infections during this time included exposure to parvovirus B19, HHV6, and EBV for which he was placed on Famvir; upper respiratory infections secondary to Mycoplasma; and an epididymitis secondary to Klebsiella, with an associated Enterobacter sinus infection. He was found to be frequently leukopenic (white cell counts ranging between 3.0 and 3.7; normal range between 4 and 9.1) with low absolute neutrophil counts (1.4, normal range 1.5 to 5.6). He also had borderline low adrenal function with decreased cortisol levels at noon (1.9 nmol/L, normal range between 2.1 and 15.7) and decreased cortisol levels in the evening (0.91 nmol/L, normal between 1.5 and 8). These may have accounted in part for his resistant fatigue. Antithyroglobulin antibodies were positive (15.1 IU/mL, normal range between 0 and 0.9) and although other autoimmune markers were negative, C4a and TFGB1 levels were elevated (C4a = 11,110 ng/mL, normal range 0–2830 ng/mL; TGFB1 level = 5340 pg/mL, normal range 344–2382 pg/mL), which are biomarkers associated with active inflammatory, mold, and autoimmune illness. Since the patient continued to suffer from recurrent infections with frequent relapses on IVIG, at age 18, the patient and his mother decided to undergo human embryonic stem cell (hESC) therapy 45, 46. The 1st set of treatments were June to July 2016 for 8 weeks at Nutech Mediworld, New Delhi, India; the 2nd set of treatments were for 4 weeks (January 2017); and the last set of treatments were June 2017 for 2 weeks. These were given through multiple routes, primarily IM and IV, although several were given through the cervical intrathecal route. The patient was simultaneously treated for Lyme disease during this same period with IV Rocephin 2 g/day, Tinidazole 1 g/day and doxycycline 100 mg BID with Bactrim DS one BID.

## Outcome and Follow‐up

Since undergoing stem cell therapy over the past one and 1/2 years, the patient has clinically stabilized with fewer sinus infections and his IgG immunoglobulin levels and subclasses have remained within normal limits. He has now decreased the use of IVIG to using Gammunex‐C every 3 months (1/2 life 30–40 days). IgG levels are drawn just before getting quarterly IVIG treatments, and they have remained within normal limits. Immunoglobulin G levels on August 2017 were normal at 944 mg/dL (normal range between 549 and 1584 mg/dL), with normal IgG subclasses 1 to 4. IgA deficiency persisted, with IgA levels remaining slightly low at 71 mg/dL (normal range between 90 and 386 mg/dL). IgA subclasses 1 and 2 also remained low during this same period of time (IgA subclass 1 = 50.1 mg/dL, normal range between 73.2 and 301.2 mg/dL; IgA subclass 2 = 8.7 mg/dL, normal range between 13.4 and 97.9 mg/dL).

Approximately 6 months post‐hESC therapy, immunoglobulin G levels remained normal at 835 mg/dL, and IgG subclasses 1–4 remained within normal limits. IgA levels had now increased to just below normal range (89 mg/dL, normal range between 90 and 386 mg/dL), and leukopenia, which was frequently seen on prior complete blood counts resolved for the first time. His last two white cell counts were 3.9 and 6.3 and were no longer in the leukopenic range.

The patient's Lyme disease symptoms have also improved. He no longer complains of significant fatigue or insomnia, and only requires low dose Adderall for his ADHD (5 mg/day) to help concentrate at school. There is mild neck and back pain, but it is positional, with no other associated joint pain or neuropathy. Recent testing for Lyme disease showed decreased Borrelia‐specific bands on the Western blot (31 kDA, i.e., Osp A, as well as a decrease in the 39 kDA band) with negative whole blood PCRs. He has remained clinically stable without relapses while off all antibiotics, and only required a seven‐day course of a cephalosporin for a sinus infection during his first year of college. Previously, he had suffered from an average of 10–15 infections per year, despite being on monthly IVIG. He is now at a high level of normal functioning, with levels of immunoglobulins remaining within normal limits before his next infusion. This is despite his quarterly immunoglobulin treatments far exceeding the normal 30–40‐day half‐life of IVIG.

## Discussion

CVID is a disorder that impairs the immune system leaving patients highly susceptible to multiple infections 13. It is associated with dysregulation of the immune system and reduced immunoglobulin/antibody levels resulting in recurrent bacterial and viral infections (e.g., sinusitis, bronchitis), with severe cases presenting with chronic diarrhea and blood count abnormalities 47. That was the case for our patient. Autoimmune disease, granulomatous disease 14, and a higher risk of malignancy (lymphoid and gastrointestinal cancers) 48 have also been associated with CVID. Although genetic mutations have been identified, the precise etiology is usually unknown.

Lyme disease has been associated with cytopathic killing of lymphocytes 49 and immunodeficiency syndromes, as long‐lived humoral immunity and immunoglobulin production has been found to be suppressed after an infection with *Borrelia burgdorferi* 44. Associated co‐infections like Mycoplasma and Bartonella 50, 51 have also been associated with immunological dysfunction, and Mycoplasmas have been found to interact with B cells 29 affecting antibody production. Hypogammaglobulinemic patients also appear to be more susceptible to colonization of mucous membranes, especially of the urogenital tract, with mycoplasmas and ureaplasmas 52 than are immunocompetent individuals. Our patient had evidence of exposure to Lyme and mycoplasma, which may have simultaneously influenced the production of antibodies. Although his sudden and severe neuropsychiatric episodes were temporally related to an initial strep infection and PANDAS, Lyme has also been reported to cause exacerbation of underlying psychiatric symptoms 53, 54 as well as being associated with autoimmune encephalopathy responsive to IVIG 55.

Other factors which could have adversely affected immune and neurological function and contributed to autoimmunity were the patient's exposure to heavy metals and mold. Mercury 31 and environmental toxins 56 are now being linked to immune dysfunction and have been linked to the worldwide increase in autoimmune disease 27, and ADHD 57. Mold toxins 28 have been associated with chronic fatigue syndrome, upper respiratory illness, and specifically gliotoxins, found in elevated levels in this patient, have been shown to be immunosuppressive 58. The patient's elevated C4a level and TGFB‐1 levels are biomarkers seen with Chronic Inflammatory Response Syndrome (CIRS) associated with mold exposure and brain injury 59. Multiple overlapping factors, including infections and toxins may have therefore contributed to the patient's clinical symptomatology.

In 2015, Wehr and her colleagues published the first study which reviewed the experience of 25 patients with CVID who underwent HSCT among fourteen centers from Europe, the United States, and Japan 60. As the cells responsible for the production of antibodies are found in the bone marrow (B cells), researchers have examined the use of bone marrow transplantation and/or stem cell transplantation (hSCT) to reverse CVID. Experience to date with hSCT is that there has been approximately a 50% cure rate, although approximately half of the patients died within 20 months after transplantation, along with a higher than expected rates of graft‐versus‐host‐disease (GvHD). Among the surviving patients, half were cured from their antibody deficiency (25% of total transplants) and the other half remained on immunoglobulin replacement despite a successful reconstitution with the donor's immune system. Our patient decided to remain on quarterly infusions of IVIG while he was in college, although his immunoglobulin levels have remained within normal range at 3 months post‐infusion, far beyond the expected half‐life of Gammaguard, which is 30–40 days.

Wehr et al. suggested 60 that there might be contributing factors to the origin of CVID outside of the hematopoietic system, which would explain the need for ongoing therapy. We have identified in this patient multiple potential overlapping etiologies of an infectious and environmental nature, which have been shown to significantly affect antibody production and immune functioning.

## Conclusion

We report the first successful improvement of CVID in a patient with Lyme disease using human embryonic stem cell therapy. Further studies are necessary to evaluate the safety and efficacy of hSCT in reversing CVID, as well as the role of infectious and environmental factors contributing to ongoing immune dysfunction in those patients failing IVIG.

## Consent

Informed consent was obtained from the patient's mother for publication of this case report.

## Disclaimer

The views expressed in this article are those of Dr Richard Horowitz, and do not represent the views of the Tick‐Borne Disease Working Group, HHS or the United States.

## Authorship

RH: provided medical care for the patient. Both authors: contributed to the writing of the paper.

## Conflict of Interest

The authors have no conflict of interests to declare.
